# Sex differences in the association between atherogenic index of plasma and progression from normoglycemia to prediabetes: evidence from a 5-year large-scale retrospective cohort study

**DOI:** 10.3389/fendo.2025.1627337

**Published:** 2025-08-12

**Authors:** Chufu Yang, Qiying Chen, Weiyan Li, Chuang Gao, Yong Han, Jiaqian Zhu

**Affiliations:** ^1^ Department of General Practice, Affiliated Hospital Group of Guangdong Medical University Shenzhen Baoan Central Hospital (Baoan Central Hospital of Shenzhen), Shenzhen, Guangdong, China; ^2^ Department of Emergency, Shenzhen Dapeng New District Kuichong People’s Hospital, Shenzhen, Guangdong, China; ^3^ Department of Emergency, Shenzhen Second People’s Hospital, The First Affiliated Hospital of Shenzhen University, Shenzhen, Guangdong, China; ^4^ Department of Neurology, The First Affiliated Hospital of Shenzhen University, Shenzhen University, Shenzhen, Guangdong, China

**Keywords:** atherogenic index of plasma, prediabetes, nonlinearity, competing risk model, sex differences

## Abstract

**Objective:**

Currently, there is limited research on the relationship between the atherogenic index of plasma (AIP) and the risk of prediabetes (pre-DM). This study aims to explore the potential link between AIP and the risk of progression from normoglycemia to pre-DM.

**Methods:**

In this retrospective cohort analysis, a total of 8,295 individuals receiving routine medical examinations at Kuichong People’s Hospital in Shenzhen between January 2018 and December 2023 were enrolled. The Cox proportional hazards regression model assessed the association between AIP and the risk of progression from normoglycemia to pre-DM, with restricted cubic splines functions used to assess non-linear relationships. Additionally, a competing risk Cox model was used, treating the progression from normoglycemia to diabetes (DM) as a competing event for pre-DM. Finally, the subgroup and sensitivity analyses confirmed the robustness of the findings.

**Results:**

After multivariable adjustment, each 0.1-unit increase in AIP was associated with an 11.5% increase in the risk of progression from normoglycemia to pre-DM [hazard ratio (HR) = 1.115; 95% confidence interval (CI): 1.065–1.167]. The competing risk Cox model showed that the sub-distribution hazard ratio for the association between AIP and the risk of pre-DM was 1.09 (95% CI: 1.04–1.14). Additionally, a non-linear association was observed in men, with an inflection point at 0.513. Below this threshold, each 0.1-unit increase in AIP was associated with an HR of 1.204 (95% CI: 1.098–1.321). In women, the relationship was linear.

**Conclusion:**

This study demonstrated that elevated AIP was positively associated with the risk of progression from normoglycemia to pre-DM, with a significant sex difference in this relationship. This provides a reference for individualized risk stratification and management strategies for different sex populations and offers new perspectives for optimizing strategies to prevent pre-DM and DM.

## Background

Prediabetes (pre-DM) refers to a metabolic condition characterized by blood glucose levels that exceed normal values yet remain below the threshold used to diagnose diabetes mellitus (DM), representing a high-risk condition for developing DM (1). The International Diabetes Federation reported that, in 2017, pre-DM affected approximately 7.7% of the global population, equivalent to around 374 million individuals (2). Projections suggest that, by 2045, the number of adults with pre-DM will rise to 548 million worldwide, representing 8.6% of the adult population (3). Each year, roughly 5%–10% of adults with pre-DM transition to overt DM, and, ultimately, about 70% of those diagnosed with pre-DM progress to DM over time (4). In addition, research has linked pre-DM to a greater likelihood of developing cardiovascular diseases, microvascular complications, certain cancers, and dementia, among other health issues (5, 6). Consequently, identifying risk factors for pre-DM and implementing targeted interventions are key steps in preventing the onset of DM and other related diseases.

Atherogenic index of plasma (AIP) was first introduced by Dobioduce and Frohlich. It is defined as the logarithm of the ratio of triglycerides (TGs) to high-density lipoprotein cholesterol (HDL-c) and serves as an important marker reflecting lipid metabolism, as well as assessing the risk of atherosclerosis and cardiovascular events (7–10). Moreover, a relationship is observed between AIP and lipoprotein particle size as well as the degree of cholesterol esterification within HDL-c (FER-HDL), both of which are closely linked to insulin resistance (IR) (10, 11). Several studies have confirmed that AIP is an effective biomarker for evaluating the risk of DM (12–14). Therefore, given that pre-DM represents an intermediate state between normal glycemia and DM, we hypothesize that elevated AIP may be closely linked to an increased risk of developing pre-DM.

Unfortunately, the current evidence on the relationship between AIP and the risk of progression from normoglycemia to pre-DM is limited and inconsistent. A cross-sectional study based on data from the U.S. National Health and Nutrition Examination Survey (NHANES) found no significant association between AIP and pre-DM risk in men, whereas a positive relationship was observed in women (15). Another cohort study conducted in China reported a positive link between AIP and pre-DM risk in the general population (16). However, both studies defined pre-DM solely based on fasting plasma glucose (FPG) levels, which may have excluded individuals meeting other diagnostic criteria, such as impaired glucose tolerance (IGT) or elevated glycated hemoglobin (HbA1c) (17). This singular definition may result in selection bias, limit the generalizability of the findings, and underestimate the true incidence of pre-DM. Additionally, most previous studies did not use a competing-risk Cox proportional hazards model to evaluate the relationship between them. However, in reality, during follow-up, once a patient is diagnosed with DM, the originally observable pre-DM events are hindered, affecting the observation of prediabetes incidence and leading to biased results. Furthermore, differences in study design, AIP value ranges, sex distribution, and adjustment for confounders exist between these studies. Therefore, the association between AIP and progression from normoglycemia to pre-DM remains unclear in Chinese. Moreover, there are significant differences in body fat percentage and distribution patterns between men and women, and the relationship between AIP and the risk of progression from normoglycemia to pre-DM may also differ by sex (18). To this end, we conducted a retrospective cohort study to investigate the association between AIP and the progression from normoglycemia to pre-DM in Chinese adults, further exploring the differences in this relationship across sex groups.

## Methods

### Study design and study population

**Figure 1:**
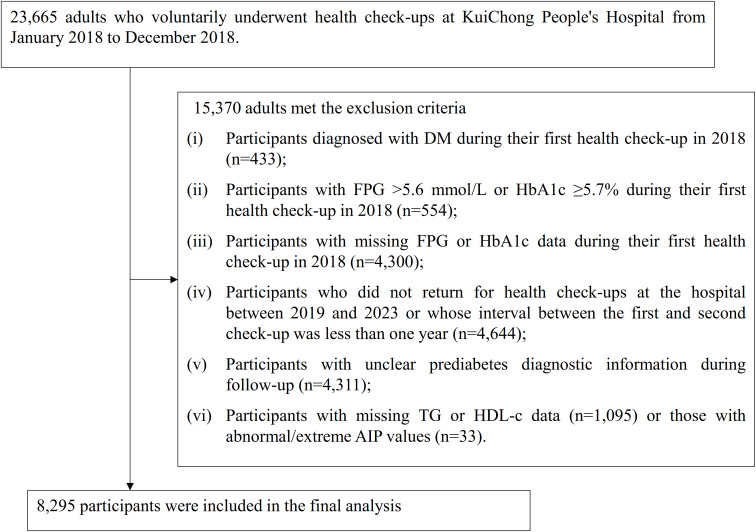
Flowchart illustrating the study participants.

Medical data were collected and analyzed retrospectively from individuals who voluntarily underwent health examinations at Kuichong People’s Hospital in Dapeng New District, Shenzhen, between January 2018 and December 2023. Initially, 23,665 adults aged 20 years or older, who completed their baseline health assessment between January and December 2018, were considered. Participants were excluded on the basis of the following criteria: (i) those with an established diagnosis of DM at baseline (n = 433); (ii) individuals with FPG >5.6 mmol/L or HbA1c ≥5.7% at the initial visit (n = 554); (iii) subjects lacking FPG or HbA1c measurements in the first examination (n = 4,300); (iv) those who either did not undergo follow-up checks at the hospital from 2019 to 2023 or whose interval between the initial and subsequent check was under 1 year (n = 4,644); (v) participants with incomplete or ambiguous pre-DM diagnostic data during follow-up (n = 4,311); and (vi) those missing TG or HDL-c values (n = 1,095), as well as individuals exhibiting extreme or invalid AIP measurements (n = 33). Ultimately, 8,295 participants were included in the final analysis. The participant screening procedure was summarized in **Figure 1**.

### Ethical approval and consent

This study received approval from the Ethics Committee of Kuichong People’s Hospital in Dapeng New District, Shenzhen (Approval No. 2024005). Given the retrospective nature of the research and the complete anonymization of all participant data, the committee granted a waiver for obtaining informed consent. Additionally, the study was conducted in full compliance with the ethical principles set forth in the Declaration of Helsinki, following all applicable ethical guidelines and regulatory requirements.

### Variables

#### Atherogenic index of plasma

The AIP was determined by calculating the base-10 logarithm of the ratio between TG and HDL-c, with both biomarkers measured in mg/dL (16). In the absence of standard risk stratification criteria for AIP, quartile-based categorization was employed, as in many previous studies, to convert continuous variables into categorical variables (19–22).

#### Definition of pre-DM

In accordance with the American Diabetes Association’s diagnostic guidelines, individuals were classified as having pre-DM if they exhibited normal glycemic status at baseline, remained free of DM throughout the follow-up, and presented with FPG values ranging from 5.6 to less than 7.0 mmol/L or HbA1c levels between 5.7% and below 6.5% (17).

#### Covariates

Selection of covariates was guided by our clinical experience as well as findings reported in earlier research (16, 23, 24). The variables used as covariates included the following: (i) continuous variables: TG, high-sensitivity C-reactive protein (Hs-CRP), body mass index (BMI), alanine aminotransferase (ALT), systolic blood pressure (SBP), FPG, aspartate aminotransferase (AST), low-density lipoprotein cholesterol (LDL-c), total cholesterol (TC), diastolic blood pressure (DBP), HDL-c, age, gamma-glutamyl transferase (GGT), HbA1c, and serum creatinine (Scr); and (ii) categorical variables: sex, hypertension (HTN), antihypertensive medication (HTN-MED), dyslipidemia (DLP), antihyperlipidemic medications (DLP-MED), smoking status, drinking status, and physical activity.

### Data collection

Trained health examination personnel performed physical assessments and gathered baseline information using standardized questionnaires. These surveys captured lifestyle variables, such as smoking and alcohol intake, along with demographic information including age, sex, and presence of HTN. Blood pressure measurements were taken using standardized mercury sphygmomanometers and fasting venous blood samples were collected after a fasting period of at least 10 hours during each examination. Biochemical parameters were analyzed using a Beckman 5800 automatic analyzer.

### Handling of missing data

This study encountered missing data for several covariates, with the number and proportion of absent values as follows: smoking status (19, 0.23%), Scr (820, 9.89%), drinking status (815, 9.83%), HTN (78, 0.94%), SBP (20, 0.24%), DBP (20, 0.24%), BMI (139,1.68%), and Hs-CRP (79, 0.95%). To mitigate potential bias arising from these missing entries, multiple imputation methods were applied (25, 26). A linear regression–based imputation model was implemented with 10 iterations, incorporating variables including sex, BMI, age, drinking status, Hs-CRP, ALT, SBP, physical activity, Scr, GGT, LDL-c, AST, smoking status, HTN, DLP-MED, and HTN-MED. Missing values were presumed to be missing at random, following accepted analytical standards (27).

### Statistical analysis

All statistical analyses were performed with R (v3.4.3) and Empower(R) software (v4.2). Statistical significance was defined as a two-tailed p-value less than 0.05. Participants were stratified on the basis of AIP quartiles, and differences between the groups were compared. For normally distributed continuous variables, results are reported as mean ± standard deviation (SD), whereas those with non-normal distributions are given as median and interquartile range. Categorical variables are described as frequencies and proportions. The analysis of variance or the Kruskal–Wallis test was utilized for continuous measures, and comparisons among groups for categorical variables were conducted using the chi-squared (χ²) test.

This investigation utilized both univariate and multivariate Cox proportional hazards regression models to examine the relationship between AIP and the risk of progression from normoglycemia to pre-DM. Three models were constructed: (i) Model I, unadjusted for any covariates; (ii) Model II, adjusted for sex and age; and (iii) Model III, which adjustment for variables including sex, BMI, age, drinking status, Hs-CRP, ALT, SBP, physical activity, smoking status, Scr, GGT, LDL-c, AST, HTN, DLP-MED, and HTN-MED. TC was excluded from the multivariate models due to multicollinearity with other predictors (**Supplementary Table S1**). Furthermore, a generalized additive model (GAM) was employed to incorporate continuous variables within the multivariable Cox proportional hazards regression model. Individuals who developed DM during the follow-up were regarded as potentially affecting the evaluation of progression from normoglycemia to pre-DM. To address this, a competing risks multivariable Cox proportional hazards model employing the Fine and Gray method was applied, treating progression from normoglycemia to DM as a competing event (28, 29).

To elucidate the potential non-linear relationship between AIP and the risk of progression from normoglycemia to pre-DM, Cox proportional hazards regression models with restricted cubic spline functions were employed separately in men, women, and the overall population. When a non-linearity was identified, a recursive algorithm was applied to determine the inflection point. Subsequently, piecewise Cox regression analyses were conducted for the intervals divided by this threshold. Model selection was based on log-likelihood ratio testing to identify the most appropriate model describing the association of AIP and progression from normoglycemic to pre-DM.

Prior research has demonstrated notable links between HTN, obesity, alcohol intake, and glucose metabolism (30–32). To confirm the robustness of our results, sensitivity analyses were performed by excluding participants with a BMI ≥28 kg/m² (33), those with alcohol consumption, or individuals diagnosed with HTN. Furthermore, to evaluate the influence of potential unmeasured confounders on the association between AIP and the risk of progression from normoglycemia to pre-DM, E-values were computed (34).

Subgroup analyses were performed using a stratified Cox proportional hazards regression models, stratifying by variables including age, SBP, DBP, physical activity, smoking, and alcohol consumption. Continuous variables such as age, SBP, and DBP were classified according to clinical cutoffs (SBP: <140 and ≥140 mmHg; age groups: <30, 30–40, 40–50, and ≥50 years; DBP: <90 and ≥90 mmHg) (35). The models were adjusted for covariates including sex, BMI, age, drinking status, Hs-CRP, ALT, SBP, physical activity, Scr, smoking status, GGT, AST, HTN, DLP-MED, LDL-c, and HTN-MED, excluding the stratification factors. Potential interactions were evaluated by comparing models with and without interaction terms using likelihood ratio tests.

## Results

### Participant characteristics

**Figure 2:**
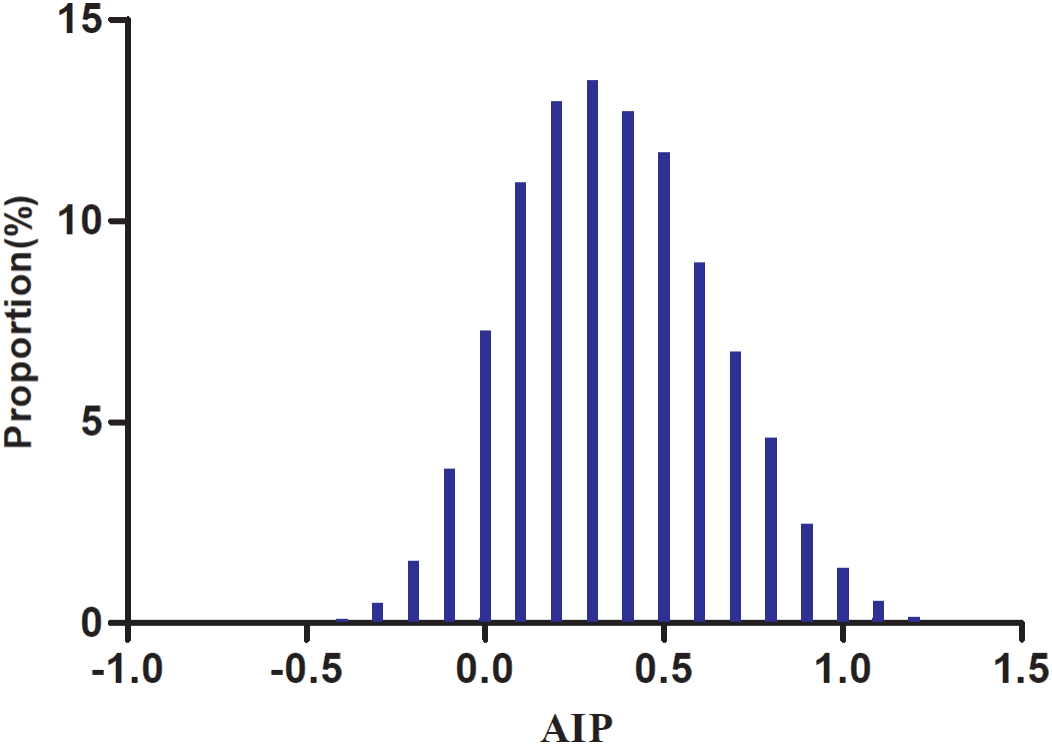
Distribution of AIP. The distribution appeared normal, spanning from −0.489 to 1.213, with a mean ± SD of 0.36 ± 0.28.

The demographic and clinical profiles of the 8,295 participants are presented in **Table 1**, with men comprising 73.79% of the study population. The AIP values followed a normal distribution, ranging from −0.489 to 1.213, with a mean (± SD) of 0.36 (± 0.28) (**Figure 2**). Participants were categorized into four groups according to AIP quartiles: Q1 (≤0.16), Q2 (0.16–0.35), Q3 (0.35–0.55), and Q4 (≥0.55). Compared with Q1, individuals in the higher AIP quartiles had higher age, DBP, SBP, BMI, TC, LDL-c, TG, FPG, AST, ALT, Hs-CRP, GGT, and HbA1c levels, as well as lower HDL-c levels. In addition, compared to Q1, those in the higher AIP quartiles showed significantly higher proportions of men, higher prevalence of HTN and DLP, and a greater proportion of sedentary individuals with low physical activity.

**Table 1:** The baseline characteristics of participants with normoglycemia.

AIP quartile	Q1 (<0.16)	Q2 (0.16–0.35)	Q3 (0.35–0.55)	Q4 (≥0.55)	P-value
N	2074	2073	2072	2076	
Age (years)	40.18 ± 8.48	41.38 ± 8.67	42.05 ± 8.60	42.38 ± 8.12	<0.001
SBP (mmHg)	111.61 ± 11.52	115.33 ± 11.08	117.82 ± 12.02	120.99 ± 12.31	<0.001
DBP (mmHg)	72.64 ± 7.56	74.92 ± 7.54	76.67 ± 7.82	78.85 ± 7.73	<0.001
BMI (kg/m^2^)	19.41 ± 3.09	20.83 ± 3.37	22.02 ± 3.62	23.32 ± 3.70	<0.001
TC (mg/dL)	183.98 ± 31.81	191.68 ± 33.45	200.34 ± 36.35	211.51 ± 38.25	<0.001
LDL-c (mg/dL)	108.35 ± 28.74	121.17 ± 31.09	129.66 ± 34.18	133.21 ± 34.79	<0.001
HDL-c (mg/dL)	62.54 ± 12.57	51.90 ± 10.10	45.38 ± 8.32	37.62 ± 6.60	<0.001
TG (mg/dL)	65.45 ± 15.66	93.16 ± 19.47	126.59 ± 24.69	205.67 ± 63.17	<0.001
FPG (mg/dL)	4.61 ± 0.39	4.73 ± 0.40	4.81 ± 0.38	4.88 ± 0.37	<0.001
AST (µ/L)	26.71 ± 8.97	28.26 ± 13.63	29.76 ± 11.98	31.65 ± 10.63	<0.001
ALT (µ/L)	29.00 (23.00–37.00)	33.00 (26.00–42.00)	37.00 (29.00–49.00)	42.00 (33.00–56.00)	<0.001
HS-CRP (mg/dL)	0.80 (0.40–1.80)	1.10 (0.50–2.30)	1.20 (0.61–2.60)	1.50 (0.80–2.70)	0.002
GGT (µ/L)	20.00 (15.00–27.00)	23.00 (18.00–33.00)	28.00 (21.00–40.00)	35.00 (26.00–50.00)	<0.001
HBA1C (%)	4.52 ± 0.25	4.59 ± 0.25	4.64 ± 0.24	4.69 ± 0.23	<0.001
Scr (µmol/L)	70.94 ± 15.78	69.99 ± 15.67	69.68 ± 15.22	70.15 ± 15.77	0.060
Sex					<0.001
Female (n, %)	1079 (52.03%)	614 (29.62%)	350 (16.89%)	131 (6.31%)	
Male (n, %)	995 (47.97%)	1,459 (70.38%)	1,722 (83.11%)	1,945 (93.69%)	
Hypertension (n, %)	96 (4.63%)	156 (7.53%)	230 (11.10%)	286 (13.78%)	<0.001
DLP (n, %)	297 (14.32%)	454 (21.90%)	619 (29.87%)	856 (41.23%)	<0.001
HTN-MED (n, %)	95 (4.58%)	146 (7.04%)	245 (11.82%)	278 (13.39%)	<0.001
DLP-MED (n, %)	110 (5.30%)	149 (7.19%)	203 (9.80%)	227 (10.93%)	<0.001
Smoking (n, %)	114 (5.50%)	152 (7.33%)	161 (7.77%)	214 (10.31%)	<0.001
Physical activity (n, %)					<0.001
Sedentary	348 (16.78%)	381 (18.38%)	454 (21.91%)	577 (27.79%)	
Low	720 (34.72%)	816 (39.36%)	775 (37.40%)	855 (41.18%)	
Moderate	725 (34.96%)	688 (33.19%)	689 (33.25%)	540 (26.01%)	
High	281 (13.55%)	188 (9.07%)	154 (7.43%)	104 (5.01%)	
Drinking status (n, %)					0.445
Ever	52 (2.51%)	48 (2.32%)	45 (2.17%)	52 (2.50%)	
Current	288 (13.89%)	257 (12.40%)	295 (14.24%)	304 (14.64%)	
Never	1,734 (83.61%)	1,768 (85.29%)	1,732 (83.59%)	1,720 (82.85%)	

Values are mean ± standard deviation or median (interquartile) or number (%).

n, number; GGT, gamma-glutamyl transferase; HDL-c, high-density lipoprotein cholesterol; TG, triglycerides; ALT, alanine aminotransferase; HbA1c, hemoglobin A1c; DLP-MED, antihyperlipidemic medications; HTN-MED, antihypertensive medication; Scr, serum creatinine; TC, total cholesterol; SBP, systolic blood pressure; DLP, dyslipidemia; FPG, fasting plasma glucose; LDL-c, low-density lipoprotein cholesterol; HS-CRP, high-sensitivity C-reactive protein; DBP, diastolic blood pressure; AST, aspartate aminotransferase; BMI, body mass index.

### The incidence of pre-DM

Over a median follow-up of 1.89 years, 322 participants (3.88%) progressed from normoglycemia to pre-DM. The incidence rates of pre-DM per 10,000 person-years for AIP quartiles Q1 to Q4 were 86.80, 206.48, 360.91, and 512.50, respectively. The overall cumulative incidence of pre-DM was 3.88%, with quartile-specific rates of 1.16% for Q1, 2.75% for Q2, 4.78% for Q3, and 6.84% for Q4. Notably, individuals in the highest AIP quartile (Q4) exhibited a significantly greater risk of progressing to pre-DM compared to those in the lowest quartile (Q1) (p for trend <0.001) (**Table 2**).

**Table 2:** Incidence of progression from normoglycemia to pre-DM (% or per 10,000 person-years).

AIP	Participants (n)	Pre-DM events (n)	Incidence rate (95% CI) (%)	Per 10,000 person-year
Total	8,295	322	3.88 (3.47–4.30)	286.70
Q1 (<0.16)	2,074	24	1.16 (0.70–1.72)	86.80
Q2 (0.16–0.35)	2,073	57	2.75 (2.05–3.45)	206.48
Q3 (0.35–0.55)	2,072	99	4.78 (3.86–5.70)	360.91
Q4 (≥0.55)	2,076	142	6.84 (5.75–7.93)	512.50
P for trend			<0.001	

n, number; CI, confidence interval.

**Figure 3:**
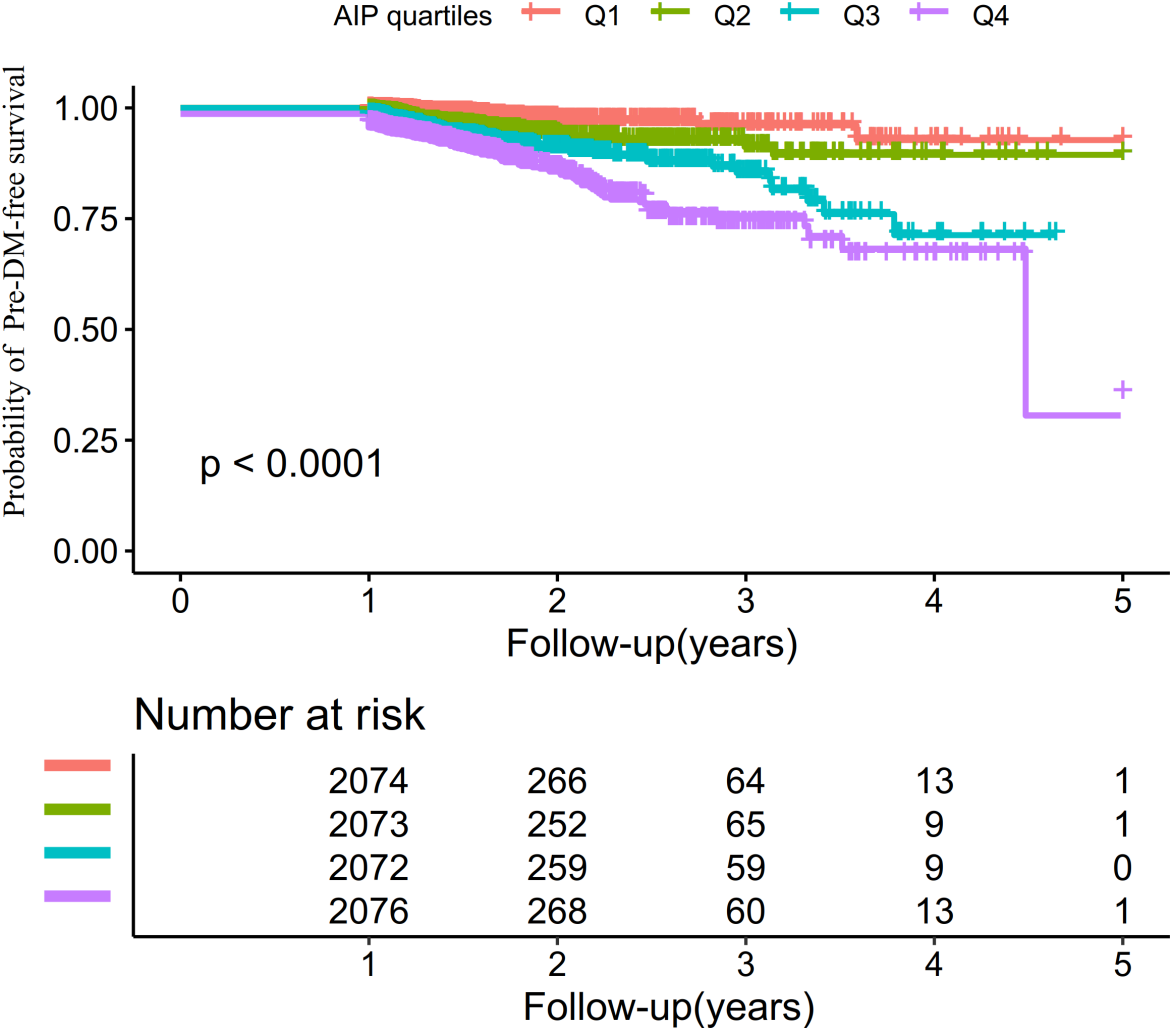
Kaplan–Meier curves illustrating pre-DM–free survival probabilities across AIP quartiles.

The Kaplan–Meier survival curves stratified by AIP quartiles (**Figure 3**) displayed the probability of pre-DM–free survival. Significant differences in pre-DM–free survival were observed among the different AIP quartiles (log-rank test, P < 0.001). The results indicated that participants in the highest quartile had the greatest risk of developing pre-DM.

### The relationship between AIP and the risk of progression from normoglycemia to pre-DM

To investigate the association between AIP and the risk of progression from normoglycemia to pre-DM, three Cox proportional hazards regression models were constructed. Model I demonstrated that each 0.1-unit elevation in AIP corresponded to a 20.7% higher risk of progressing from normoglycemia to pre-DM (HR = 1.207; 95% CI: 1.162–1.254). Following adjustment for demographic factors, Model II also revealed a significant link, with a 12.4% increased risk per 0.1-unit rise in AIP (HR = 1.124; 95% CI: 1.077–1.174). In Model III, which accounted for a comprehensive set of potential confounders, the association persisted, revealing an 11.5% heightened risk of pre-DM for every 0.1-unit increment in AIP (HR = 1.115; 95% CI: 1.065–1.167) (**Table 3**).

**Table 3:** The relationship between AIP and the risk of progression from normoglycemia to pre-DM.

Exposure	Model I (HR, 95% CI) P	Model II (HR, 95% CI) P	Model III (HR, 95% CI) P	Model IV (HR, 95% CI) P
AIP (per 0.1 unit)	1.207 (1.162, 1.254) < 0.001	1.124 (1.077, 1.174) < 0.001	1.115 (1.065, 1.167) < 0.001	1.113 (1.061, 1.167) < 0.001
AIP quartile				
Q1	Ref	Ref	Ref	Ref
Q2	2.385 (1.480, 3.842) < 0.001	1.748 (1.081, 2.825) 0.023	1.727 (1.064, 2.803) 0.027	1.612 (0.986, 2.635) 0.057
Q3	4.164 (2.666, 6.505) < 0.001	2.533 (1.603, 4.001) < 0.001	2.374 (1.491, 3.779) < 0.001	2.266 (1.410, 3.642) < 0.001
Q4	5.906 (3.832, 9.103) < 0.001	3.014 (1.916, 4.742) < 0.001	2.812 (1.763, 4.486) < 0.001	2.664 (1.650, 4.301) < 0.001
P for trend	<0.001	<0.001	<0.001	<0.001

Model I: We did not adjust other covariates.

Model II: We adjust sex and age.

Model III: We adjust sex, BMI, drinking status, age, Hs-CRP, ALT, SBP, physical activity, smoking status, Scr, GGT, LDL-c, AST, hypertension, DLP-MED, and HTN-MED.

Model IV: We adjusted sex, drinking status, age (smooth), BMI (smooth), Hs-CRP (smooth), ALT (smooth), SBP (smooth), physical activity, smoking status, Scr (smooth), GGT (smooth), LDL (smooth), hypertension, AST (smooth), DLP-MED, and HTN-MED.

HR, hazard ratio; Ref, reference; CI, confidence interval.

Additionally, AIP was categorized on the basis of quartile distribution and incorporated again into the Cox proportional hazards regression models. With the lowest quartile (Q1) serving as the reference, multivariate adjustment revealed HRs for developing pre-DM of 1.727 (95% CI: 1.064–2.803) in Q2, 2.374 (95% CI: 1.491–3.779) in Q3, and 2.812 (95% CI: 1.763–4.486) in Q4. These findings suggest that, relative to Q1, individuals in the Q2 group had a 72.7% higher risk of progression from normoglycemia to pre-DM, whereas those in Q3 and Q4 exhibited 137.4% and 181.2% increased risks, respectively (**Table 3**, Model III).

### The competitive risk multivariate cox proportional hazards regression results

**Table 4** summarizes the findings from the competitive risk multivariate Cox proportional hazards regression analysis, where the development from normoglycemia to DM was considered a competing event in the progression to pre-DM. In the crude model, AIP showed a significant positive link between AIP and the risk of progression from normoglycemia to pre-DM, with a sub-distribution hazard ratio (SHR) of 1.21 (95% CI: 1.16–1.25) per 0.1-unit AIP increase. After adjusting for age and sex in Model I, the SHR for the association between AIP (per 0.1-unit) and pre-DM risk was 1.12 (95% CI: 1.08–1.17). In Model II, which adjusted for potential confounders including sex, BMI, age, drinking status, Hs-CRP, ALT, SBP, physical activity, smoking status, Scr, GGT, LDL-c, AST, HTN, DLP-MED, and HTN-MED, the positive association between AIP (per 0.1 unit) and the risk of pre-DM remained, with an SHR of 1.09 (95% CI: 1.04–1.14).

**Table 4:** The relationship between AIP and the progression from normoglycemia to pre-DM in different competing risk models.

Exposure	Crude model (SHR, 95% CI, P)	Model I (SHR, 95% CI, P)	Model II (SHR, 95% CI, P)
AIP (per 0.1 unit)	1.21 (1.16, 1.25) < 0.001	1.12 (1.08, 1.17) < 0.001	1.09 (1.04, 1.14) < 0.001
AIP quartiles			
Q1	Ref.	Ref.	Ref.
Q2	2.38 (1.48, 3.84) < 0.001	1.75 (1.08, 2.83) 0.023	1.61 (0.99, 2.61) 0.055
Q3	4.16 (2.67, 6.50) < 0.001	2.53 (1.60, 4.00) < 0.001	2.18 (1.37, 3.47) 0.001
Q4	5.91 (3.83, 9.10) < 0.001	3.01 (1.92, 4.74) < 0.001	2.29 (1.44, 3.65) < 0.001
P for trend	<0.001	<0.001	<0.001

Crude Model: We did not adjust other covariates.

Model I: We adjust sex and age.

Model II: We adjust sex, drinking status, BMI, age, Hs-CRP, ALT, SBP, physical activity, smoking status, Scr, GGT, LDL-c, AST, hypertension, DLP-MED, and HTN-MED.

SHR, sub-distribution hazard ratio; Ref, reference; CI, confidence interval.

When AIP was examined as a categorical variable, comprehensive adjustment in Model II indicated that, using the Q1 as the reference, the SHR for Q2 was 1.61 (95% CI: 0.99–2.61). The SHRs for participants in Q3 and Q4 were 2.18 (95% CI: 1.37–3.47) and 2.29 (95% CI: 1.44–3.65), respectively. Analysis of the confidence intervals revealed that, compared with the reference group, the increased risk of pre-DM in the Q2 was not statistically significant, whereas significant risk elevations were observed in Q3 and Q4. The overall upward trend from Q1 to Q4 was consistent with the results observed when AIP was analyzed as a continuous variable (P for trend <0.05) (**Table 4**, Model II).

### Sensitivity analysis

To confirm the robustness of our results, multiple sensitivity analyses were performed. Initially, a GAM was applied to incorporate continuous covariates as smooth functions. The findings, presented in **Table 3** (Model IV), were largely aligned with those obtained from the fully adjusted Model III. Specifically, each 0.1-unit rise in AIP corresponded to an 11.3% increase hazard of progression from normoglycemia to pre-DM (HR = 1.113; 95% CI: 1.061–1.167). Further sensitivity analysis restricted the sample to individuals with a BMI under 28 kg/m², where the positive association between AIP (per 0.1-unit increase) and pre-DM risk remained significant after adjusting for confounders (HR = 1.128; 95% CI: 1.075–1.184). Similarly, excluding participants with HTN produced similar results, showing an HR of 1.145 (95% CI: 1.089–1.204) per 0.1-unit increase in AIP. Lastly, among never drank, the relationship persisted significantly, with an HR of 1.119 (95% CI: 1.063–1.178) (**Table 5**).

**Table 5:** The relationship between AIP and the progression from normoglycemia to pre-DM in different sensitivity analyses.

Exposure	Crude model (SHR, 95% CI, P)	Model I (SHR, 95% CI, P)	Model II (SHR, 95% CI, P)
AIP (per 0.1 unit)	1.128 (1.075, 1.184) < 0.001	1.145 (1.089, 1.204) < 0.001	1.119 (1.063, 1.178) < 0.001
AIP quartiles			
Q1	Ref	Ref	Ref
Q2	1.655 (1.002, 2.734) 0.049	2.078 (1.214, 3.560) 0.008	1.652 (0.970, 2.814) 0.065
Q3	2.695 (1.676, 4.333) < 0.001	3.127 (1.856, 5.269) < 0.001	2.429 (1.459, 4.045) < 0.001
Q4	2.912 (1.800, 4.712) < 0.001	3.628 (2.147, 6.129) < 0.001	2.908 (1.739, 4.863) < 0.001
P for trend	<0.001	<0.001	<0.001

HR, hazard ratio; CI: confidence interval.

Model I involved a sensitivity analysis of participants with BMI <28 kg/m^2^ (n = 7,856). Sex, age, drinking status, Hs-CRP, ALT, SBP, physical activity, smoking status, Scr, GGT, LDL-c, AST, hypertension, DLP-MED, and HTN-MED were adjusted.

Model II involved sensitivity analyses after excluding participants with hypertension (N = 7,527). Sex, BMI, age, drinking status, Hs-CRP, ALT, SBP, physical activity, smoking status, Scr, GGT, LDL-c, AST, DLP-MED, and HTN-MED were adjusted.

Model III involved sensitivity analyses of participants with never drank (N = 6,954). Sex, BMI, age, Hs-CRP, ALT, SBP, physical activity, smoking status, Scr, GGT, LDL-c, AST, hypertension, DLP-MED, and HTN-MED were adjusted.

Furthermore, the calculated E-value of 1.47 was higher than the relative risk linked to AIP and potential unmeasured confounders (estimated at 1.34). This suggests that unknown or unmeasured confounding factors are unlikely to have a significant impact on the relationship between AIP and the risk of progression from normoglycemia to pre-DM. These sensitivity analyses reinforce the credibility and stability of our results.

### Subgroup analysis

In both predefined and exploratory subgroup analyses (**Supplementary Table S2**), no significant interactions were detected between AIP and variables such as age, SBP, smoking, DBP, physical activity, and drinking status (all interaction P-values > 0.05). These findings indicate that these factors did not significantly affect or alter the link between AIP and the risk of progression from normoglycemia to pre-DM. In contrast, sex appears to significantly modify their relationship (P for interaction <0.001). Specifically, the relationship between elevated AIP and the risk of progression from normoglycemia to pre-DM was stronger in women (HR = 1.425; 95% CI: 1.234–1.646), whereas this association was comparatively weaker in men (HR = 1.090; 95% CI: 1.039–1.143).

### Non-linear relationship between AIP and the risk of progression from normoglycemia to pre-DM in sex subgroups and in all participants

**Figure 4:**
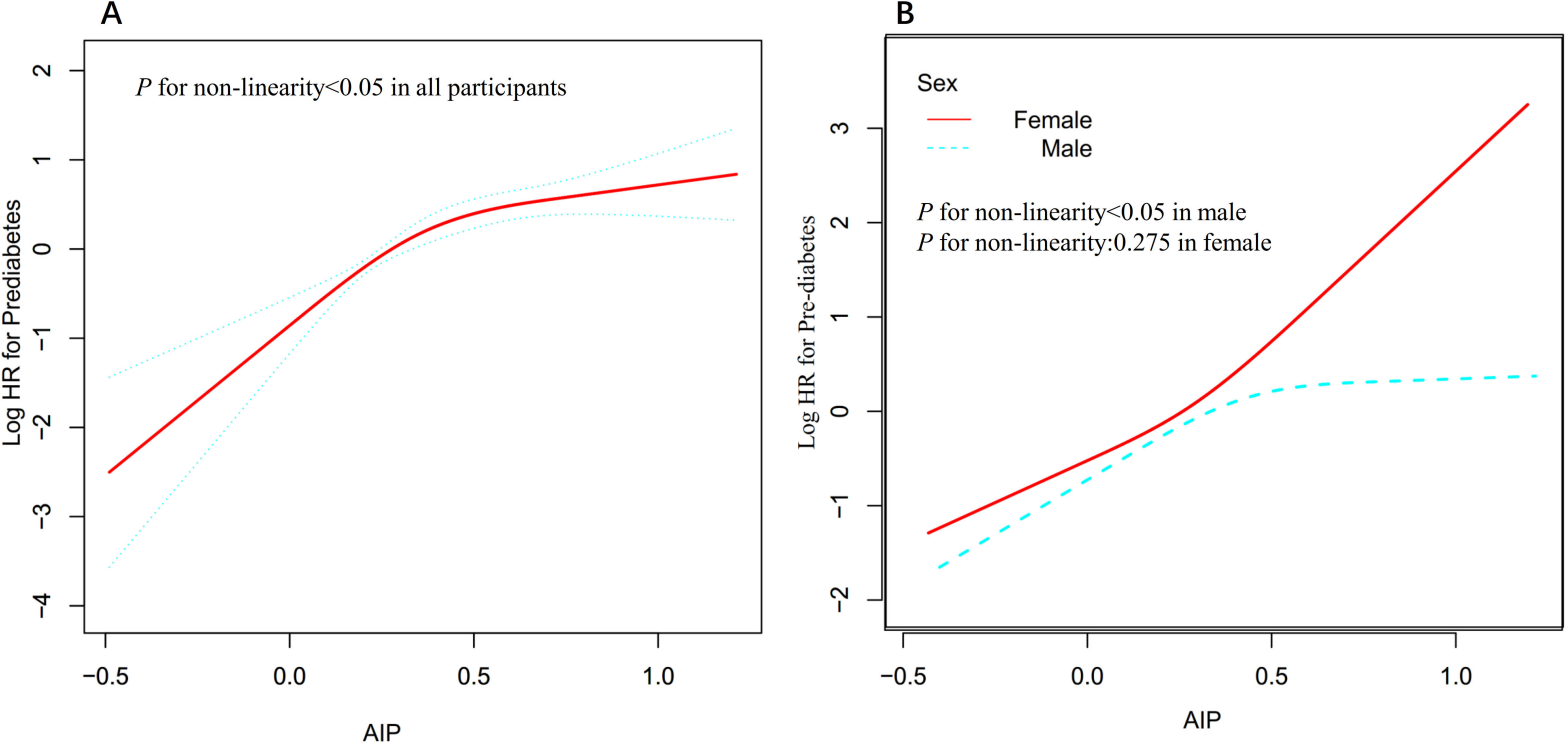
Non-linear relationship between AIP and the risk of progression from normoglycemia to pre-DM all participants **(A)** and in sex subgroups **(B)**.

Utilizing a Cox proportional hazards regression model with restricted cubic spline functions, a non-linear association was identified between AIP and the risk of progression from normoglycemia to pre-DM in men and all participants (P for non-linearity <0.05, **Figure 4**). A recursive algorithm determined an inflection point at an AIP value of 0.513. To better characterize this relationship, a two-piece Cox regression model was applied to estimate the HR and CI on either side of this threshold. Below the inflection point, each 0.1-unit increase in AIP was associated with HRs of 1.204 (95% CI: 1.098–1.321) in men and 1.213 (95% CI: 1.109–1.327) in all participants, whereas, above the inflection point, no statistically significant associations were observed in either group (**Table 6**). In contrast, no non-linear relationship between AIP and the risk of progression from normoglycemia to pre-DM was found in women (p for non-linearity ≥0.05, **Figure 4**).

**Table 6:** The result of two-piecewise Cox regression model in sex subgroups and all participants.

	Female	Male	All participants
Fitting model by two-piecewise Cox regression			
Inflection points of AIP	0.513	0.513	0.513
<0.513 (per 0.1 unit)	1.473 (1.130, 1.920) 0.004	1.204 (1.098, 1.321) < 0.001	1.213 (1.109, 1.327) < 0.001
≥0.513 (per 0.1 unit)	1.356 (0.940, 1.955) 0.103	1.006 (0.910, 1.113) 0.908	1.027 (0.941, 1.121) 0.559
P for log-likelihood ratio test	0.760	0.028	0.006

Note 1: Above model adjusted for sex, BMI, drinking status, age, Hs-CRP, ALT, SBP, physical activity, Scr, smoking status, AST, GGT, LDL-c, hypertension, DLP-MED, and HTN-MED.

Note 2: The model was not adjusted for sex as a covariate when analyses were stratified by sex.

## Discussion

This retrospective cohort study demonstrates an independent positive association between AIP and the risk of progression from normoglycemia to pre-DM. Additionally, a saturation effect curve with an inflection point at an AIP value of 0.513 was observed in men. In contrast, the relationship in women was found to be linear.

AIP is recognized as a superior marker reflecting dyslipidemia and has been widely used to assess the risk of atherosclerosis (9, 36, 37). In addition, AIP has also demonstrated significant value in evaluating the risk of DM (12–14). However, current research examining the relationship between AIP and the progression from normoglycemia to pre-DM remains limited and inconsistent. A cross-sectional study based on data from the U.S. National Health and NHANES reported that, after adjusting for confounders, each 1-unit increase in AIP was associated with a 4.96-fold higher risk of pre-DM and DM in female participants (OR = 4.96; 95% CI: 2.68–9.18), whereas no significant association was observed in male participants (15). Another cohort study with an average follow-up of 3.12 years found that, in the general population, each 1-unit increase in AIP was linked to a 41% higher risk of developing pre-DM (HR = 1.41; 95% CI: 1.31–1.52) (16).

Our study complements existing literature by supporting the hypothesis that elevated AIP is positively associated with the progression from normoglycemia to pre-DM. Compared with previous studies that predominantly defined pre-DM based solely on FPG, we combined FPG and HbA1c to provide a more comprehensive definition of pre-DM. In addition, our study specifically focused on the dynamic transition from normoglycemia to pre-DM and treated AIP as both a categorical and a continuous variable. These methodological approaches reduced information loss and enabled a more precise quantification of the association between AIP and the outcome, thereby further enhancing the clinical applicability and accuracy of our conclusions. Although most earlier studies relied on traditional linear regression analyses, we also employed restricted cubic spline functions to investigate the potential non-linear dose-response relationship between AIP and pre-DM risk. Additionally, we innovatively introduced a competing risk model to fully account for the possibility of multiple endpoint events during follow-up, a methodological improvement that allowed risk assessment to better approximate real-world clinical scenarios. Finally, a series of sensitivity analyses and subgroup analyses were conducted to verify the robustness of our findings.

Notably, this study primarily evaluated the association between AIP and the risk of pre-DM among adults with normoglycemia. As pre-DM represents an intermediate stage in the progression from normoglycemia to type 2 DM (T2DM), the association between AIP and pre-DM is mainly applicable to diseases characterized by IR and dyslipidemia, such as T2DM and potentially gestational DM (GDM), because AIP effectively reflects these metabolic changes (38, 39). In contrast, type 1 DM (T1DM) is primarily an autoimmune disease characterized by β-cell destruction, and alterations in lipid profiles have relatively little impact on its pathogenesis (40). Thus, our findings are unlikely to be applicable to T1DM. Although our results cannot be directly extrapolated to pregnant women, previous studies suggest that AIP may be useful in identifying women at risk for GDM, which warrants further investigation (41). Our study has demonstrated a significant association between AIP and the progression from normoglycemia to pre-DM, providing a valuable reference for the prevention of pre-DM, T2DM, and their complications. Incorporating AIP into routine clinical assessments may assist healthcare professionals in identifying high-risk individuals at an early stage, thereby optimizing risk stratification and management strategies. Reducing AIP through regular physical activity and dietary modifications, such as lowering TG levels or increasing HDL-c levels, may ultimately decrease the incidence of DM and alleviate the public health burden.

The specific mechanisms underlying the association between elevated AIP and the risk of progression from normoglycemia to pre-DM were not yet fully understood but may be related to disorders of glucose metabolism. Higher AIP typically reflects elevated TG levels or reduced HDL-C levels. Elevated TG levels increase free fatty acids, inducing lipotoxicity that interferes with insulin signaling in pancreatic α-cells, leading to excessive glucagon secretion and subsequent IR (42). IR further exacerbates the increase in TG levels, creating a vicious cycle of metabolic dysfunction (43). Additionally, low HDL-C levels weaken its antioxidant and anti-inflammatory protective effects on pancreatic β-cells, promoting cholesterol accumulation within β-cells, which triggers cellular dysfunction and apoptosis, thereby inhibiting normal insulin secretion (44–46). These potential mechanisms help provide a pathophysiological explanation for the relationship between AIP and the development of pre-DM.

Subgroup analysis showed that sex modified the association between AIP and the risk of progression from normoglycemia to pre-DM. In women, elevated AIP was more strongly associated with an increased risk of pre-DM, whereas this association was weaker in men. This sex difference may be explained by several factors. First, men and women differ in physiological metabolism, including patterns of body fat distribution and metabolic characteristics. Women typically have a higher percentage of body fat and different fat distribution compared to men, which may affect lipid metabolism and IR (18). Second, differences in sex hormone levels may also influence lipid metabolism and insulin sensitivity, thereby affecting the association between AIP and pre-DM risk (47). Meanwhile, the baseline characteristics were compared between men and women, and it was found that women had lower BMI, SBP, GGT, LDL-c, DBP, and AST levels, as well as a lower proportion of sedentary behavior, all of which are closely related to the risk of pre-DM or DM (**Supplementary Table S3**) (48–51). Therefore, in men, the association between AIP and the risk of pre-DM is relatively attenuated because of higher levels of these risk factors, whereas, in women, this association is relatively strengthened because these risk factors are present at lower levels. Furthermore, when participants were stratified by AIP quartiles, the multivariable-adjusted model showed that, compared to the first quartile, HR for pre-DM risks were 1.727, 2.374, and 2.812 in the Q2, Q3, and Q4, respectively. This indicates that HR values show a marked upward trend from Q1 to Q4, whereas this trend plateaus at Q4, suggesting a possible non-linear relationship between them. To verify this, we used a Cox proportional hazards regression model incorporating a restricted cubic spline function and found a non-linear association between AIP and the risk of progression from normoglycemia to pre-DM in men, with an inflection point at 0.513. Below this threshold, every 0.1-unit increase in AIP was associated with a 20.4% increased risk of pre-DM; however, the association was no longer statistically significant above 0.513. In contrast, women exhibited a linear association. This may also be one of the reasons for the sex differences in the association between AIP and the risk of progression from normoglycemia to pre-DM. This finding provides a reference for individualized risk stratification in populations of different sexes and offers a new perspective for optimizing the prevention strategies of pre-DM and DM. Among men, maintaining AIP below 0.513 and further reducing AIP through dietary interventions and lifestyle modifications may significantly decrease the risk of progression from normoglycemia to pre-DM. In women, continuously lowering AIP levels may help reduce the incidence of pre-DM.

The relationship between AIP and the risk of progression from normoglycemia to pre-DM is non-linear, which may be closely related to multiple physiological and metabolic mechanisms. AIP, as the logarithmic transformation of the ratio of TG to HDL-C, can reflect the severity of IR (13). Lower levels of AIP indicate lower IR, which has a significant role in regulating blood glucose levels. Within this range, a slight increase in AIP can significantly affect glucose metabolic homeostasis, leading to an increased risk of progression from normoglycemia to pre-DM. However, when AIP exceeds a certain threshold, the effect of IR on glucose metabolism tends to saturate and further increases in AIP do not lead to a significant increase in pre-DM risk (52, 53). This “plateau” effect may explain the non-linear relationship between AIP and pre-DM risk.

This study demonstrates several notable strengths. First, the relationship between AIP and the progression from normoglycemia to pre-DM was assessed by considering AIP both as a continuous measure and as quartile-based categories. Second, a non-linear association between AIP and pre-DM risk in men was characterized, including identification of a threshold effect, whereas a linear pattern was observed in women—an important advancement in understanding sex-specific differences. Third, missing data were addressed using multiple imputations, which improved statistical efficiency and minimized bias due to incomplete information on covariates. Finally, the robustness of the results was evaluated through extensive sensitivity analyses, which involved modeling continuous covariates as smooth terms with GAM, employing competing risk models, and reanalyzing the AIP–pre-DM association after excluding participants with BMI exceeding 28 kg/m², those with HTN, or individuals with a history of alcohol use.

However, several limitations were noted. First, the study population was limited to Chinese individuals, which restricted the generalizability of the findings to other ethnicities or regions, indicating the need for further validation in diverse populations. Second, only baseline measurements of AIP and other related parameters were conducted, and longitudinal changes in AIP over time were not assessed. Future studies were recommended to focus on collecting more comprehensive longitudinal data on AIP fluctuations. Additionally, due to the retrospective cohort design, adjustments for potential confounders-including postprandial blood glucose, dietary characteristics, allergic conditions, family history of DM, genetic susceptibility, and insulin levels were limited. However, E-values were calculated to evaluate the potential impact of unmeasured confounding factors, and it was found that such factors were unlikely to have significantly influenced the results. Future studies should include a broader range of relevant variables, such as lifestyle factors and lipid-lowering medication use, to enable a more comprehensive analysis of the association between AIP and the progression from normoglycemia to pre-DM, thereby further validating our findings. Finally, it should be emphasized that, as an observational study, an independent association between AIP and pre-DM was identified; however, causality could not be established.

## Conclusion

This study demonstrates that elevated AIP is positively associated with the risk of progressing from normoglycemia to pre-DM and that there are significant sex-specific differences in this association. Furthermore, in men, a saturation effect curve was observed, with an inflection point at 0.513. These findings suggest the need for personalized interventions: in men, reducing AIP below 0.513 through dietary interventions and lifestyle modifications may significantly lower the risk of developing pre-DM, whereas, in women, ongoing monitoring and effective reduction of AIP levels may help reduce the risk of pre-DM. This study provides valuable insights for optimizing clinical management and prevention strategies for pre-DM and DM, particularly in terms of sex-specific AIP management approaches.

## Data Availability

The original contributions presented in the study are included in the article/**Supplementary Material**. Further inquiries can be directed to the corresponding author.
