# Supplementary material for: Sex differences in the association between atherogenic index of plasma and progression from normoglycemia to prediabetes: evidence from a 5-year large-scale retrospective cohort study
Source: Front Endocrinol (Lausanne). 2025 Aug 12;16:1627337. doi: 10.3389/fendo.2025.1627337 (PMC12378059; doi:10.3389/fendo.2025.1627337)
Supplement: Supplementary file 1 [file Table1.docx]

**Sex differences in the association between atherogenic index of plasma and progression from normoglycemia to prediabetes: evidence from a 5-Year large-scale retrospective cohort study.**

**Running title:** AIP and prediabetes

**Chufu Yang^1^*, Qiying Chen^1^*, Weiyan Li^1^*, Chuang Gao^2^, Yong Han^3^**^†^**, Jiaqian Zhu^4^**^†^

^1^ Department of General Practice, Affiliated Hospital Group of Guangdong Medical University Shenzhen Baoan Central Hospital (Baoan Central Hospital of Shenzhen), Shenzhen 518100, Guangdong Province, China.

^2^ Department of Emergency, Shenzhen Dapeng New District Kuichong People's Hospital, Shenzhen 518000, Guangdong Province, China.

^3^ Department of Emergency, Shenzhen Second People's Hospital, The First Affiliated Hospital of Shenzhen University, Shenzhen 518000, Guangdong Province, China.

**^4^** School of Medicine, Shenzhen University, The First Affiliated Hospital of Shenzhen University, Shenzhen 518000, Guangdong Province, China.

**Chufu Yang^1^*, Qiying Chen^1^* and Weiyan Li^1^*** contributed equally.

*** Correspondence:**

Jiaqian Zhu

Department of Neurology, The First Affiliated Hospital of Shenzhen University

3002 Sungang West Road, Futian District,

Shenzhen 518000,

Guangdong Province,

China.

Email: zhujiaqian1122@163.com

***Corresponding author**

Yong Han

Department of Emergency, Shenzhen Second People's Hospital

No.3002 Sungang West Road, Futian District,

Shenzhen 518035,

Guangdong Province,

China.

Email: Hanyong511023@163.com

**Table S1 Collinearity screening**

|  | Step 1 | Step 2 |
| --- | --- | --- |
| AIP | 1.5 | 1.5 |
| Age | 1.2 | 1.2 |
| Sex | 1.7 | 1.5 |
| Hypertension | 4.4 | 4.4 |
| SBP | 3.1 | 3.1 |
| DBP | 3.1 | 3.1 |
| BMI | 1.5 | 1.5 |
| Smoking | 1 | 1 |
| TC | 10.1 | NA |
| LDL-c | 9.6 | 1.1 |
| TG | 2 | 1.2 |
| FPG | 1.2 | 1.2 |
| ALT | 1.7 | 1.7 |
| AST | 2.2 | 2.2 |
| Hs-CRP | 1 | 1 |
| Physical Activity | 1.1 | 1.1 |
| Scr | 1 | 1 |
| Drinking | 1 | 1 |

Variables excluded by collinearity screening: TC

**Table S2 Stratified associations between AIP and the progression from normoglycemia to pre-DM by age, sex, SBP, physical activity, smoking, and drinking**

| Characteristic | No of participants | HR (95%CI) P value P for interaction |
| --- | --- | --- |
| Age(years) |  | 0.220 |
| <30 | 446 | 1.164 (0.896, 1.513) 0.256 |
| 30-40 | 3199 | 1.164 (1.071, 1.264) <0.001 |
| 40-50 | 3131 | 1.139 (1.064, 1.220) <0.001 |
| ≥50 | 1519 | 1.048 (0.971, 1.131) 0.228 |
| Sex |  | <0.001 |
| Female | 2174 | 1.425 (1.234, 1.646) <0.001 |
| Male | 6121 | 1.090 (1.039, 1.143) <0.001 |
| SBP (mmHg) |  | 0.053 |
| <140 | 7772 | 1.150 (1.096, 1.206) <0.001 |
| ≥140 | 523 | 1.018 (0.908, 1.142) 0.758 |
| DBP (mmHg) |  | 0.099 |
| <90 | 7669 | 1.129 (1.076, 1.186) <0.001 |
| ≥90 | 626 | 1.010 (0.892, 1.144) 0.872 |
| Physical Activity |  | 0.961 |
| **Sedentary** | 1715 | 1.110 (1.020, 1.207) 0.015 |
| **Light activity** | 3113 | 1.123 (1.045, 1.206) 0.002 |
| Moderate activity | 2622 | 1.119 (1.036, 1.209) 0.004 |
| Vigorous activity | 718 | 1.071 (0.907, 1.264) 0.421 |
| Smoking |  | 0.822 |
| No | 7654 | 1.117 (1.064, 1.171) <0.001 |
| Yes | 641 | 1.100 (0.968, 1.249) 0.144 |
| Drinking |  | 0.943 |
| Ever | 197 | 1.154 (0.937, 1.421) 0.177 |
| Current | 1144 | 1.109 (0.994, 1.239) 0.065 |
| Never | 6954 | 1.114 (1.060, 1.171) <0.001 |

Note 1: Above model adjusted for sex, BMI, age, drinking status, Hs-CRP, ALT, SBP, physical activity, smoking status, Scr, GGT, LDL-c, AST, hypertension, DLP-MED, HTN-MED.

Note 2: In each case, the model is not adjusted for the stratification variable.

HR, Hazard ratios; CI: confidence, Ref: reference.

**Table S3 Comparison of baseline characteristics between males and females**

| Characteristics | Female | Male | P-value |
| --- | --- | --- | --- |
| N | 2174 | 6121 |  |
| Age (years) | 40.21 ± 8.30 | 41.96 ± 8.54 | <0.001 |
| SBP (mmHg) | 108.68 ± 11.31 | 119.20 ± 11.33 | <0.001 |
| DBP (mmHg) | 70.76 ± 7.53 | 77.55 ± 7.38 | <0.001 |
| BMI (kg/m^2^) | 23.87 ± 3.74 | 26.61 ± 3.47 | <0.001 |
| TC (mg/dL) | 188.75 ± 33.76 | 199.77 ± 37.02 | <0.001 |
| LDL-c (mg/dL) | 109.08 ± 30.46 | 128.08 ± 33.36 | <0.001 |
| AST (u/L) | 23.89 ± 7.17 | 30.94 ± 12.26 | <0.001 |
| ALT (u/L) | 27.05 ± 11.05 | 43.53 ± 20.63 | <0.001 |
| HS-CRP (mg/dL) | 2.92 ± 5.98 | 2.09 ± 4.36 | <0.001 |
| GGT (u/L) | 21.46 ± 14.25 | 36.99 ± 26.87 | <0.001 |
| Smoking (n, %) | 153 (7.04%) | 488 (7.97%) | 0.161 |
| Physical Activity (n, %) |  |  | <0.001 |
| Sedentary | 531 (24.43%) | 1229 (20.08%) |  |
| Low | 852 (39.19%) | 2314 (37.80%) |  |
| Moderate | 646 (29.71%) | 1996 (32.61%) |  |
| High | 145 (6.67%) | 582 (9.51%) |  |
| Drinking status (n, %) |  |  | 0.321 |
| Ever | 52 (2.39%) | 145 (2.37%) |  |
| current | 279 (12.83%) | 865 (14.13%) |  |
| Never | 1843 (84.77%) | 5111 (83.50%) |  |

Values are mean ± standard deviation or median (interquartile) or number (%). GGT, gamma-glutamyl transferase; HDL-c, high-density lipoprotein cholesterol; TG, triglycerides; ALT, alanine aminotransferase; TC, total cholesterol; SBP, systolic blood pressure; DLP, dyslipidemia; FPG, fasting plasma glucose; LDL-c, low-density lipoprotein cholesterol; HS-CRP, high-sensitivity C-reactive protein; DBP, diastolic blood pressure; AST, aspartate aminotransferase; BMI, body mass index.
